# Bibliographical Notices

**Published:** 1838-03-28

**Authors:** 


### BIBLIOGRAPHICAL NOTICES.

The Philosophy of Health; *or, an Exposition of the Physical and Mental Constitution of Man, with a View to the Promotion of Human Longevity and Happiness; by* Southwood Smith, M. D. Physician to the London Fever Hospital, to the Eastern Dispensary, and to the Jews Hospital. Vol. II. London, 1837. 8vo. pp. 448.

It is not quite four years since Dr. Combe's excellent little volume, on the "Principles of Physiology" appeared amongst us, and the results of its great and well-merited popularity, have been, if we mistake not, of a most wholesome and extensive nature. Works of the description of Dr. Combe's and of the one now before us, are calculated in every respect to command the attention of a large and highly intelligent class of readers, and in the end are productive of incalculable benefit, operating sensibly and happily on the community in general. They familiarize man with the history and elements of his own creation; they cause him to investigate the structure and functions of his own frame; they satisfy that healthful curiosity concerning his organization which is inherent in every one of God's creatures; and in the gratification of this desire, regarding his physical state, they enlarge and ennoble his moral and intellectual aptitude and condition. We have ever held as utter ambominations all Domestic Treatises on Medicine. These Invalid Vade-Mecums, invariably, do infinite harm; they are the manuals of persons who are totally ignorant of the structure and offices of the machine they are heedlessly tampering with. But we conceive that no rational being can be found who will raise similar objections to Dr. Southwood Smith's "Philosophy of Health." The practitioner of medicine should especially encourage such publications; for the more accurate and universal is the knowledge of anatomy and physiology the higher will be the station of his science in the public view, the more exalted will be the individual rank of the physician, more implicit will be the respect paid to his prescriptions and opinions, and as the process by which the human frame is formed and maintained becomes familiar, empiricism will gradually decline into utter extinction. Quackery flourishes, Dr. Johnson has somewhere observed, because nine-tenths of mankind are ignorant of medicine, meaning doubtless of its elements.

The great end of living, is to live well; i. e. in a state of bodily, and, as a consequence, mental integrity. Now we are continually exposed to the influence of external agents, which militate strongly against this condition of perfect sanity, and which by a series of aggressions, as insidious as formidable, engender innumerable maladies.

Definite perceptions of the construction of the human edifice, and of the varied and complex actions of the animal economy, and an acquaintance with the nature and operation of these deleterious agents, must inevitably lead to the most felicitous results towards the conservation of health and happiness, and necessarily to the perfection of the physical and intellectual man.

All this, we contend, concerns more or less remotely, every member of the human family, and most nearly the moralist, the legislator, the jurist, and particularly those to whose charge the education of a future generation is to be confided.

The grossest errors are daily committed by the judge and the barrister in questions involving property and even life itself, and simply from inexcusable ignorance of the first principles of the science of being.

It is an acknowledged fact, and one which the social theory admits, that the impressions made during the second epoch of infancy—the period extending from the seventh month to the second year—are vivid and momentous; and that the influences then made on the plastic creature at the moment its sentient faculties are budding, control powerfully and lastingly the future character of the individual. To whose care is the being at this time confided, and from whom are these impressions derived—what is this source of all future good or ill? *The Mother!* Yet what precautions are instituted in the education of our females to adapt them to so vital and delicate a trust; and what qualifications do they possess to enable them to discharge it with fidelity; what knowledge have they of the tender structure committed to their tutilage? *None.* On the contrary, their habits, opinions and prejudices peculiarly unfit them for the trust, and are indeed positive disqualifications. The nuptial blessing, while it invests the girl with the title and offices of the matron, disposes no physiological knowledge essential to the high duties she may soon be called upon to assume.

Is there not then an implied moral obligation that an acquaintance with the fundamental principles of the science of organization and life, should form not an unimportant portion in the education of every female?

Dr. Smith, who is a disciple of the late Jeremy Bentham, contends very justly that *between happiness and longevity an intimate relation prevails.* Enjoyment, he thinks, is not only the end of life, but is alone the condition which endears existence to us. If a life has been totally exempt from suffering either mentally or physical, if it has embraced every enjoyment, and excluded every care, it must of necessity be a long and prosperous one. The mind and the body mutually react on each other: Mental disquietude can only influence the health through the physical condition, and thus affect the duration of life. Longevity and happiness are then in general co-existent. If, on the contrary, continued and intense pain and misfortune have been the share, life becomes scarcely tolerable, and soon ceases.

The announcement in the decalogue—"That thy days may be long in the land which the Lord thy God hath given thee"—as the highest reward, would appear to sanction Dr. Smith's theory, and would countenance the idea that a faithful discharge of our duties will be crowned by a protracted life of enjoyment.

In all countries the average duration of existence is longest among that class which enjoys the greatest proportion of comforts, and, of course, is less liable to disease. All statistics prove this conclusively. Villermé tells us that in the poorer arondissements of Paris the deaths are 100 per cent. greater than in the wealthier quarters of the metropolis. The same results obtain in the departments. The deaths among the pauper population and in the prisons are double that of the usual average. Dr. Gouveneur Emerson, in a valuable paper published a few years since in the American Journal of the Medical Sciences, states the mortality among the black inhabitants of this city, to be nearly twice as great as that of the whites. In the same volume, (pp. 111–2–3,) Dr. Smith proves pretty satisfactorily that the "seven ages" of man are not mere arbitrary divisions, or the offspring of poetical fancy, but are founded in sober reason and fact. That the periods of infancy, childhood, adolescence, and old age cannot be extended any more than they can be abridged. Yet are we continually meeting with persons who, though actually in the prime of manhood, appear to have experienced the vicissitudes of at least four-score winters, whilst others, who in the course of nature we might naturally anticipate finding in all the completeness of senility, "sans teeth, sans eyes, sans taste, sans every thing," enjoy their faculties in all their pristine vigor. How can these facts be explained if our premises be correct. Dr. Smith's solution is as consoling as it is ingenious. He shall speak for himself.

"The duration of the periods of infancy, childhood, boyhood, and adolescence, is fixed by a certain number of years. Nothing can stay, nothing retard, the succession of each. Alike incapable of any material protraction is the period of old age. It follows that every year by which the term of human existence is extended is really added to the period of mature age; the period when the organs of the body have attained their full growth, and put forth their full strength; when the physical organization has acquired its utmost perfection; when the senses, the feelings, the emotions, the passions, the affections, are in the highest degree acute, intense, and varied; when the intellectual faculties, completely unfolded and developed, carry on their operations with the greatest vigour, soundness, and continuity: in a word, when the individual is capable of receiving and of communicating the largest amount of the highest kind of enjoyment.

"A consideration more full of encouragement, more animating, there cannot be. The extension of human life, in whatever mode and degree it may be possible to extend it, is the protraction of that portion of it, and only of that portion of it, in which the human being is capable of RECEIVING AND OF COMMUNICATING THE LARGEST MEASURE OF THE NOBLEST KIND OF ENJOYMENT." (P. 114.)

Having thus cursorily glanced at the objects of attraction, which works of this description possess for the general reader, and having briefly and incidentally alluded to the importance of the first principles of anatomy and physiology to every intelligent individual, and which should entitle it to a place in the curriculum of study in all colleges and schools, we shall close these general remarks with a few words to the professional reader.

The utter neglect in this country of *Hygiène* by practitioners of medicine is lamentable, and we had almost said criminal. It is a mortifying fact that in most of our medical schools no especial provision is made for it, and indeed where it is announced as constituting part of a chair, we suspect that, in general, it is merely titular, bloating the blazoned insignia of the professional dignitary, and realizing no intrinsic benefit to the pupil.

Every physician obtains in the course of time some hygienic experience, but this is too often dearly purchased at the expense of the comfort, health and even lives of many, who, by the timely application of common sense preventives might have survived long and happily. These are melancholy truths, but at the same time are undeniable, and demand the serious and immediate attention of the physician and philanthropist.

If we could consult our inclination we should present our readers with a pretty full analysis of Dr. Southwood Smith's excellent volume, and gratify them by transferring to our pages many passages of striking beauty with which the work abounds. This wish our limits forbid us to indulge, and we must confine ourselves to a few remarks on the general execution of the design.

It is seldom our lot to meet with a work as unexceptionable in every respect as the present. Our author has treated of a very interesting subject in a very entertaining and edifying manner. The vast mass of invaluable information contained in this volume is conveyed with singular perspicuity, and with a richness of illustration rarely encountered. The style is remarkable for its clearness, precision and conciseness, at the same time that the language is distinguished for its beauty and copiousness, and very often rises into positive eloquence. Whilst the most polished reader would be charmed by its terseness and elegance, a child would be enabled, from its simplicity, to comprehend very fully every sentence. This we know is high praise and may be deemed extravagant, but the reader, on perusal, will find it merited.

The present volume, with the previous one, treats of the structure and functions of organic life; the succeeding one will embrace those of animal existence, and proceed to the examination of the action of physical agents on the human economy.

The illustrations, which are numerous, are distinguished for fidelity and neatness of execution. A glossary, explanatory of the technical terms, which by the way are used with rather a lavish hand, would make the work more generally useful.

CRANIA AMERICANA: *or, a Comparative View of the Skulls of various Aboriginal Nations of North and South America, with an Essay on the Varieties of the Human Species, and on the American Race in particular. Illustrated by Sixty Plates, and a coloured Map. By* SAMUEL GEORGE MORTON, M. D., M. A. P. S. &c. &c. *Philadelphia: John Fuller.*

We have received several specimen sheets of this work, which we have great pleasure in bringing before the notice of the profession. It will be a most valuable, and certainly a very original addition to the scientific literature of our country, being, as the author announces in the prospectus, "a record of a series of facts of no small novelty and interest, both to the general and scientific reader, and derived from a field of inquiry, hitherto in a great measure unexplored." It will contain lithographic illustrations of the skulls of our North and South American Indian nations, including an exposition of the distortions,caused by mechanical contrivances among various tribes, with a particular notice of the Crania from the mounds and caves of the western section of this country. These illustrations will form the prominent features of the work, not to the exclusion however of a discussion of a variety of interesting topics which they naturally suggest. The lithographs, which we have seen, are admirably executed: there are to be about seventy in all, each containing a drawing of a cranium of the natural size. The text is to embrace between two and three hundred imperial quarto pages—from what is before us, we are justified in speaking in high praise of the style of execution.

The work is to be published by Mr. John Fuller, Philadelphia, for the author; is, it is announced, in a state of considerable forwardness, and will be delivered to subscribers on the first of October, 1838. Price of subscription, twenty dollars: the work will be delivered to subscribers *only*.

